# Supplementary material for: Dynamic Modeling of Streptococcus pneumoniae Competence Provides Regulatory Mechanistic Insights Into Its Tight Temporal Regulation
Source: Front Microbiol. 2018 Jul 24;9:1637. doi: 10.3389/fmicb.2018.01637 (PMC6066662; doi:10.3389/fmicb.2018.01637)
Supplement: Supplementary file 1 [file Table_1.PDF]

Table S1

Sequences of primers used in this study

Primers	Sequence
MB26	CTTTTTTCGTAGGAAAAGAAAATCAAGGATGATTCTTGAAATCCTCATCTCCCC
MB27	CGCTATTTTGTCTGTTTGCCGAATA
MB28	GGGGAGATGAGGATTTCAAGAATCATCCTTGATTTTCTTTTCCTACGAAAAAAG
MB29	TACAACATCTTCATTTTCAAGTAACATACTCTTCG
MB54	TTCCATGGCTAGAAAGGGGAAAATTATGATTAAAG
MB56	TTCCATGGCTAGAAAGGGGAAAATTATGATTAAAG
MB57	GTTTACTATCCGTACCCATTAGTAAGAAAAAAGAAAAGGAGTATTTGATTATG
MB58	CCGGATCCCCTCAACAAGAAATAAACCCCCG
