# Supplementary material for: Dynamic Modeling of Streptococcus pneumoniae Competence Provides Regulatory Mechanistic Insights Into Its Tight Temporal Regulation
Source: Front Microbiol. 2018 Jul 24;9:1637. doi: 10.3389/fmicb.2018.01637 (PMC6066662; doi:10.3389/fmicb.2018.01637)
Supplement: Supplementary file 2 [file Table_2.PDF]

# Table S2

Rate reaction laws

| Reaction | Equation | Kinetic rate law |
|---|---|---|
| ComAB synthesis | $\beta_{ComAB} + vmax_{ComAB} \frac{[(ComE{\sim}P)_D]}{[(ComE{\sim}P)_D] + K_{ComE{\sim}P\_AB} * \left(1 + \frac{[ComE]^e}{{Ki_{ComE\_AB}}^e}\right)}$ | Competitive inhibition |
| ComAB degradation | $\gamma_{ComAB} * [ComAB]$ | Mass action |
| pre-CSP synthesis | $\beta_{ComCDE} + vmax_{ComCDE} \frac{[(ComE{\sim}P)_D]}{[(ComE{\sim}P)_D] + K_{ComE{\sim}P} * \left(1 + \frac{[ComE]^e}{{Ki_{ComE}}^e}\right)}$ | Competitive inhibition |
| pre-CSP maturation and CSP export | $\varepsilon * [ComAB] * [preCSP]$ | Mass action |
| pre-CSP degradation | $\gamma_{preCSP} * [preCSP]$ | Mass action |
| CSP degradation | $\gamma_{CSP} * [CSP]$ | Mass action |
| ComE synthesis | $\beta_{ComCDE} + vmax_{ComCDE} \frac{[(ComE{\sim}P)_D]}{[(ComE{\sim}P)_D] + K_{ComE{\sim}P} * \left(1 + \frac{[ComE]^e}{{Ki_{ComE}}^e}\right)}$ | Competitive inhibition |
| ComE degradation | $\gamma_{ComE} * [ComE]$ | Mass action |
| ComD synthesis | $\beta_{ComCDE} + vmax_{ComCDE} \frac{[(ComE{\sim}P)_D]}{[(ComE{\sim}P)_D] + K_{ComE{\sim}P} * \left(1 + \frac{[ComE]^e}{{Ki_{ComE}}^e}\right)}$ | Competitive inhibition |
| Dimerization of ComD | $k_{on\_D} * [ComD]^2$ | Mass action |
| ComD dimer dissociation | $k_{off\_D} * [(ComD)_D]$ | Mass action |
| Basal autophosphorylation of ComD | $\alpha_{auto} * [(ComD)_D]$ | Mass action |
| CSP-induced autophosphorylation of ComD | $vmax_{act} * \frac{[CSP]}{[CSP] + K_{act}} * [(ComD)_D]$ | Michaelis-Menten |
| ComD degradation | $\gamma_{ComD} * [ComD]$ | Mass action |
| Degradation of the inactive form of the CSP-bound dimer of ComD | $\gamma_{ComD} * [(ComD\_consumed)_D]$ | Mass action |
| ComE transphosphorylation | $\lambda * [ComE]^2 * [(ComD_{act})_D]$ | Mass action |
| ComE~P dephosphorylation | $\rho * [(ComE{\sim}P)_D]$ | Mass action |
| ComX synthesis as an inactive form | $vmax_{ComX} * \frac{[(ComE{\sim}P)_D]^x}{[(ComE{\sim}P)_D]^x + {K_{ComX}}^x}$ | Hill function (cooperativity) |
| ComW synthesis | $vmax_{ComW} * \frac{[(ComE{\sim}P)_D]^w}{[(ComE{\sim}P)_D]^w + {K_{ComW}}^w}$ | Hill function (cooperativity) |
| ComX activation | $\omega_1 * [ComW] * [ComX_{ina}]$ | Mass action |
| ComW degradation | $\gamma_{ComW} * [ComW]$ | Mass action |
| Inactive form of ComX degradation | $\gamma_{ComX} * [ComX_{ina}]$ | Mass action |
| Active form of ComX degradation | $\gamma_{ComX} * [ComX_{act}]$ | Mass action |
| SsbB synthesis | $vmax_{SsbB} * \frac{[ComX_{act}]^s}{[ComX_{act}]^s + {K_{SsbB}}^s}$ | Hill function (cooperativity) |
| SsbB degradation | $\gamma_{SsbB} * [SsbB]$ | Mass action |
| DprA synthesis | $vmax_{DprA} * \frac{[ComX_{act}]^d}{[ComX_{act}]^d + {K_{DprA}}^d}$ | Hill function (cooperativity) |
| DprA degradation | $\gamma_{DprA} * [DprA]$ | Mass action |
| Formation of the DprA-ComE~P complex | $2 * k_{on\_DprA\_EP} * [DprA]^2 * [(ComE{\sim}P)_D]$ | Mass action |
| Degradation of the DprA-ComE~P complex | $\gamma_{DprA} * [(DprA\_ComE{\sim}P)_D]$ | Mass action |
| ComZ synthesis | $vmax_{ComZ} * \frac{[ComX_{act}]^z}{[ComX_{act}]^z + {K_{ComZ}}^z}$ | Hill function (cooperativity) |
| ComZ degradation | $\gamma_{ComZ} * [ComZ]$ | Mass action |
| ComZ and ComW interaction | $\omega_2 * [ComZ] * [ComW_{act}]$ | Mass action |
