# Supplementary material for: Dynamic Modeling of Streptococcus pneumoniae Competence Provides Regulatory Mechanistic Insights Into Its Tight Temporal Regulation
Source: Front Microbiol. 2018 Jul 24;9:1637. doi: 10.3389/fmicb.2018.01637 (PMC6066662; doi:10.3389/fmicb.2018.01637)
Supplement: Supplementary file 3 [file Table_3.pdf]

Table S3

New or modified reactions and ODE equations for the alternative models

**Interaction between ComW and a late *com* gene product ComZ prevents ComW activity**	
**New reactions**	
Synthesis of ComZ	$ComX_{act} \rightarrow ComX_{ina} + ComZ$
Inhibition of active form of ComW	$ComW_{act} + ComZ \rightarrow ComW_{ina}$
**New ODEs**	

$$\frac{d[ComZ]}{dt} = vmax_{ComZ} * \frac{[ComX_{act}]^z}{[ComX_{act}]^z + K_{ComZ}{}^z} - \omega_2 * [ComZ] * [ComW_{act}] - \gamma_{ComZ} * [ComZ]$$

$$\frac{d[ComW_{act}]}{dt} = vmax_{ComW} \frac{[(ComE{\sim}P)_D]^w}{[(ComE{\sim}P)_D]^w + K_{ComW}{}^w} - \omega_2 * [ComZ] * [ComW_{act}] - \gamma_{ComW} * [ComW_{ina}]$$

$$\frac{d[ComW_{ina}]}{dt} = \omega_2 * [ComZ] * [ComW_{act}] - \gamma_{ComW} * [ComW_{ina}]$$

$$[ComW]_{total} = [ComW_{ina}] + [ComW_{act}]$$

**Competition between ComW and a late *com* gene product ComZ for the inactive form of ComX impairs the formation of the active form of ComX**	
**New reactions**	
Synthesis of ComZ	$ComX_{act} \rightarrow ComX_{ina} + ComZ$
Inhibition of ComX activation	$ComX_{ina} + ComW + ComZ \rightarrow ComX_{act} + ComW + ComZ$
**New ODEs**	

$$\frac{d[ComZ]}{dt} = vmax_{comZ} * \frac{[ComX_{act}]^z}{[ComX_{act}]^z + K_{comZ}{}^z} - \gamma_{ComZ} * [ComZ]$$

$$\frac{d[ComX_{act}]}{dt} = vmax_{ComX_{act}} * \frac{[ComW_{act}]^j}{[ComW_{act}]^j + K_{ComX_{act}}{}^j} * \left(1 - \frac{[ComZ]^k}{[ComZ]^k + Ki_{ComX_{act}\_ComZ}{}^k}\right) * [ComX_{ina}] - vmax_{DprA} * \frac{[ComX_{act}]^d}{[ComX_{act}]^d + K_{DprA}{}^d} - vmax_{SsbB} * \frac{[ComX_{act}]^s}{[ComX_{act}]^s + K_{SsbB}{}^s} - vmax_{ComZ} * \frac{[ComX_{act}]^z}{[ComX_{act}]^z + K_{ComZ}{}^z} - \gamma_{ComX} * [ComX_{act}]$$

$$\frac{d[ComX_{ina}]}{dt} = vmax_{ComX} * \frac{[(ComE{\sim}P)_D]^x}{[(ComE{\sim}P)_D]^x + K_{ComX}{}^x} + vmax_{DprA} * \frac{[ComX_{act}]^d}{[ComX_{act}]^d + K_{DprA}{}^d} + vmax_{SsbB} * \frac{[ComX_{act}]^s}{[ComX_{act}]^s + K_{SsbB}{}^s} + vmax_{ComZ} * \frac{[ComX_{act}]^z}{[ComX_{act}]^z + K_{ComZ}{}^z} - vmax_{ComX_{act}} * \frac{[ComW_{act}]^j}{[ComW_{act}]^j + K_{ComX_{act}}{}^j} * \left(1 - \frac{[ComZ]^k}{[ComZ]^k + Ki_{ComX_{act}\_ComZ}{}^k}\right) * [ComX_{ina}] - \gamma_{ComX} * [ComX_{ina}]$$

**Competition between the active form of ComX and a late *com* gene product ComZ for RNA polymerase binding**	
**New reactions**	
Synthesis of ComZ	$ComX_{act} \rightarrow ComX_{ina} + ComZ$
Synthesis of SsbB	$ComX_{act} + ComZ \rightarrow ComX_{ina} + SsbB + ComZ$
Synthesis of DprA	$ComX_{act} + ComZ \rightarrow ComX_{ina} + DprA + ComZ$
**New ODEs**	

$$\frac{d[ComZ]}{dt} = \beta_{ComZ} + vmax_{ComZ} * \frac{[ComX_{act}]^z}{[ComX_{act}]^z + K_{ComZ}{}^z}\left(1 - \frac{[ComZ]^i}{[ComZ]^i + Ki_{ComZ\_ComZ}{}^i}\right) - \gamma_{ComZ} * [ComZ]$$

$$\frac{d[SsbB]}{dt} = vmax_{SsbB} * \frac{[ComX_{act}]^s}{[ComX_{act}]^s + K_{SsbB}{}^s} * \left(1 - \frac{[ComZ]^i}{[ComZ]^i + Ki_{ComZ\_SsbB}{}^i}\right) - \gamma_{SsbB} * [SsbB]$$

$$\frac{d[DprA]}{dt} = vmax_{DprA} * \frac{[ComX_{act}]^d}{[ComX_{act}]^d + K_{DprA}{}^d} * \left(1 - \frac{[ComZ]^u}{[ComZ]^u + Ki_{ComZ\_DprA}{}^u}\right) - 2 * k_{on\_DprA\_EP} * [DprA]^2 * [(ComE{\sim}P)_D] - \gamma_{DprA} * [DprA]$$

$$\frac{d[ComX_{act}]}{dt} = \omega_1 * [ComW] * [ComX_{ina}] - vmax_{DprA} * \frac{[ComX_{act}]^d}{[ComX_{act}]^d + K_{DprA}{}^d}\left(1 - \frac{[ComZ]^u}{[ComZ]^u + Ki_{ComZ\_DprA}{}^u}\right) - vmax_{SsbB} * \frac{[ComX_{act}]^s}{[ComX_{act}]^s + K_{SsbB}{}^s}\left(1 - \frac{[ComZ]^i}{[ComZ]^i + Ki_{ComZ\_SsbB}{}^i}\right) - vmax_{ComZ} * \frac{[ComX_{act}]^z}{[ComX_{act}]^z + K_{ComZ}{}^z}\left(1 - \frac{[ComZ]^i}{[ComZ]^i + Ki_{ComZ\_ComZ}{}^i}\right) - \gamma_{ComX} * [ComX_{act}]$$

$$\frac{d[ComX_{ina}]}{dt} = vmax_{ComX} * \frac{[(ComE{\sim}P)_D]^x}{[(ComE{\sim}P)_D]^x + K_{ComX}{}^x} - \omega_1 * [ComW] * [ComX_{ina}] + vmax_{DprA} * \frac{[ComX_{act}]^d}{[ComX_{act}]^d + K_{DprA}{}^d}\left(1 - \frac{[ComZ]^u}{[ComZ]^u + Ki_{ComZ\_DprA}{}^u}\right) + vmax_{SsbB} * \frac{[ComX_{act}]^s}{[ComX_{act}]^s + K_{SsbB}{}^s}\left(1 - \frac{[ComZ]^i}{[ComZ]^i + Ki_{ComZ\_SsbB}{}^i}\right) + vmax_{ComZ} * \frac{[ComX_{act}]^z}{[ComX_{act}]^z + K_{ComZ}{}^z}\left(1 - \frac{[ComZ]^i}{[ComZ]^i + Ki_{ComZ\_ComZ}{}^i}\right) - \gamma_{ComX} * [ComX_{ina}]$$

Inhibition of the active form of ComX by a late *com* gene product ComZ

New reactions

Synthesis of ComZ	$ComX_{act} \rightarrow ComX_{ina} + ComZ$
Inhibition of ComX	$ComX_{act} + ComZ \rightarrow ComX_{ina}$

New ODEs

$$\frac{d[ComZ]}{dt} = vmax_{ComZ} * \frac{[ComX_{act}]^z}{[ComX_{act}]^z + K_{ComZ}{}^z} - \omega_2 * [ComZ] * [ComX_{act}] - \gamma_{ComZ} * [ComZ]$$

$$\frac{d[ComX_{act}]}{dt} = \omega_1 * [ComW] * [ComX_{ina}] - \omega_2 * [ComX_{act}] * [ComZ] - vmax_{DprA} * \frac{[ComX_{act}]^d}{[ComX_{act}]^d + K_{DprA}{}^d} - vmax_{SsbB} * \frac{[ComX_{act}]^s}{[ComX_{act}]^s + K_{SsbB}{}^s} - vmax_{ComZ} * \frac{[ComX_{act}]^z}{[ComX_{act}]^z + K_{ComZ}{}^z} - \gamma_{ComX} * [ComX_{act}]$$

$$\frac{d[ComX_{ina}]}{dt} = vmax_{ComX} * \frac{[(ComE{\sim}P)_D]^x}{[(ComE{\sim}P)_D]^x + K_{ComX}{}^x} + vmax_{DprA} * \frac{[ComX_{act}]^d}{[ComX_{act}]^d + K_{DprA}{}^d} + vmax_{SsbB} * \frac{[ComX_{act}]^s}{[ComX_{act}]^s + K_{SsbB}{}^s} + vmax_{ComZ} * \frac{[ComX_{act}]^z}{[ComX_{act}]^z + K_{ComZ}{}^z} + \omega_2 * [ComX_{act}] * [ComZ] - \omega_1 * [ComW] * [ComX_{ina}] - \gamma_{ComX} * [ComX_{ina}]$$

Interaction between ComW and an early *com* gene product ComZ impairs ComW activity

New reactions

Synthesis of ComZ	$(ComE{\sim}P)_D \rightarrow (ComE{\sim}P)_D + ComZ$
Inhibition of active ComW	$ComW_{act} + ComZ \rightarrow ComW_{ina}$

New ODEs

$$\frac{d[ComZ]}{dt} = vmax_{ComZ} * \frac{[ComE{\sim}P]^Z}{[ComE{\sim}P]^Z + K_{ComZ}{}^Z} - \omega_2 * [ComZ] * [ComW_{act}] - \gamma_{ComZ} * [ComZ]$$

$$\frac{d[ComW_{act}]}{dt} = vmax_{ComW} * \frac{[(ComE{\sim}P)_D]^w}{[(ComE{\sim}P)_D]^w + K_{ComW}{}^w} - \omega_2 * [ComZ] * [ComW_{act}] - \gamma_{ComW} * [ComW_{act}]$$

$$\frac{d[ComW_{ina}]}{dt} = \omega_2 * [ComZ] * [ComW_{act}] - \gamma_{ComW} * [ComW_{ina}]$$

$$[ComW_{total}] = [ComW_{ina}] + [ComW_{act}]$$

**Competition between ComW and an early *com* gene product ComZ for the inactive form of ComX impairs the formation of the active form of ComX**	
**New reactions**	
Synthesis of ComZ	$(ComE{\sim}P)_D \rightarrow (ComE{\sim}P)_D + ComZ$
Inhibition of ComX activation	$ComX_{ina} + ComW + ComZ \rightarrow ComX_{act} + ComW + ComZ$

New ODEs

$$\frac{d[ComZ]}{dt} = vmax_{ComZ} * \frac{[ComE{\sim}P]^{Z}}{[ComE{\sim}P]^{Z} + {K_{ComZ}}^{Z}} - \gamma_{ComZ} * [ComZ]$$

$$\frac{d[ComX_{act}]}{dt} = vmax_{ComX_{act}} * \frac{[ComW_{act}]^{j}}{[ComW_{act}]^{j} + {K_{ComX_{act}}}^{j}} * \left(1 - \frac{[ComZ]^{k}}{[ComZ]^{k} + {Ki_{ComX_{act}\_ComZ}}^{k}}\right) * [ComX_{ina}] - vmax_{DprA} * \frac{[ComX_{act}]^{d}}{[ComX_{act}]^{d} + {K_{DprA}}^{d}} - vmax_{SsbB} * \frac{[ComX_{act}]^{s}}{[ComX_{act}]^{s} + {K_{SsbB}}^{s}} - \gamma_{ComX} * [ComX_{act}]$$

$$\frac{d[ComX_{ina}]}{dt} = vmax_{ComX} * \frac{[(ComE{\sim}P)_D]^{x}}{[(ComE{\sim}P)_D]^{x} + {K_{ComX}}^{x}} + vmax_{DprA} * \frac{[ComX_{act}]^{d}}{[ComX_{act}]^{d} + {K_{DprA}}^{d}} + vmax_{SsbB} * \frac{[ComX_{act}]^{s}}{[ComX_{act}]^{s} + {K_{SsbB}}^{s}} - \gamma_{ComX} * [ComX_{ina}] - vmax_{ComX_{act}} * \frac{[ComW_{act}]^{j}}{[ComW_{act}]^{j} + {K_{ComX_{act}}}^{j}} * \left(1 - \frac{[ComZ]^{k}}{[ComZ]^{k} + {Ki_{ComX_{act}\_ComZ}}^{k}}\right) * [ComX_{ina}]$$

**Competition between the active form of ComX and an early *com* gene product ComZ for RNA polymerase binding**	
New Reactions	
Synthesis of ComZ	$(ComE{\sim}P)_D \rightarrow (ComE{\sim}P)_D + ComZ$
Synthesis of SsbB	$ComX_{act} + ComZ \rightarrow ComX_{ina} + SsbB + ComZ$
Synthesis of DprA	$ComX_{act} + ComZ \rightarrow ComX_{ina} + DprA + ComZ$

New ODEs

$$\frac{d[ComZ]}{dt} = \beta_{ComZ} + vmax_{ComZ} * \frac{[ComE{\sim}P]^{Z}}{[ComE{\sim}P]^{Z} + {K_{ComZ}}^{Z}} - \gamma_{ComZ} * [ComZ]$$

$$\frac{d[SsbB]}{dt} = vmax_{SsbB} * \frac{[ComX_{act}]^{s}}{[ComX_{act}]^{s} + {K_{SsbB}}^{s}} * \left(1 - \frac{[ComZ]^{i}}{[ComZ]^{i} + {Ki_{ComZ\_SsbB}}^{i}}\right) - \gamma_{SsbB} * [SsbB]$$

$$\frac{d[DprA]}{dt} = vmax_{DprA} * \frac{[ComX_{act}]^{d}}{[ComX_{act}]^{d} + {K_{DprA}}^{d}} * \left(1 - \frac{[ComZ]^{u}}{[ComZ]^{u} + {Ki_{ComZ\_DprA}}^{u}}\right) - 2 * k_{on\_DprA\_EP} * [DprA]^{2} * [(ComE{\sim}P)_D] - \gamma_{DprA} * [DprA]$$

$$\frac{d[ComX_{act}]}{dt} = \omega_1 * [ComW] * [ComX_{ina}] - vmax_{DprA} * \frac{[ComX_{act}]^{d}}{[ComX_{act}]^{d} + {K_{DprA}}^{d}}\left(1 - \frac{[ComZ]^{u}}{[ComZ]^{u} + {Ki_{ComZ\_DprA}}^{u}}\right) - vmax_{SsbB} * \frac{[ComX_{act}]^{s}}{[ComX_{act}]^{s} + {K_{SsbB}}^{s}}\left(1 - \frac{[ComZ]^{i}}{[ComZ]^{i} + {Ki_{ComZ\_SsbB}}^{i}}\right) - \gamma_{ComX} * [ComX_{act}]$$

$$\frac{d[ComX_{ina}]}{dt} = vmax_{ComX} * \frac{[(ComE{\sim}P)_D]^{x}}{[(ComE{\sim}P)_D]^{x} + {K_{ComX}}^{x}} - \omega_1 * [ComW] * [ComX_{ina}] + vmax_{DprA} * \frac{[ComX_{act}]^{d}}{[ComX_{act}]^{d} + {K_{DprA}}^{d}}\left(1 - \frac{[ComZ]^{u}}{[ComZ]^{u} + {Ki_{ComZ\_DprA}}^{u}}\right) + vmax_{SsbB} * \frac{[ComX_{act}]^{s}}{[ComX_{act}]^{s} + {K_{SsbB}}^{s}}\left(1 - \frac{[ComZ]^{i}}{[ComZ]^{i} + {Ki_{ComZ\_SsbB}}^{i}}\right) - \gamma_{ComX} * [ComX_{ina}]$$

**Inhibition of the active form of ComX by an early *com* gene product ComZ**	
**New reactions**	
Synthesis of ComZ	$(ComE{\sim}P)_D \rightarrow (ComE{\sim}P)_D + ComZ$
Inhibition of ComX	$ComX_{act} + ComZ \rightarrow ComX_{ina}$

New ODEs

$$\frac{d[ComZ]}{dt} = vmax_{comZ} * \frac{[ComE{\sim}P]^Z}{[ComE{\sim}P]^Z + K_{ComZ}{}^Z} - \omega_2 * [ComX_{act}] * [ComZ] - \gamma_{ComZ} * [ComZ]$$

$$\frac{d[ComX_{act}]}{dt} = \omega_1 * [ComW] * [ComX_{ina}] - \omega_2 * [ComX_{act}] * [ComZ] - vmax_{DprA} * \frac{[ComX_{act}]^d}{[ComX_{act}]^d + K_{DprA}{}^d} - vmax_{SsbB} * \frac{[ComX_{act}]^s}{[ComX_{act}]^s + K_{SsbB}{}^s} - \gamma_{ComX} * [ComX_{act}]$$

$$\frac{d[ComX_{ina}]}{dt} = vmax_{ComX} * \frac{[(ComE{\sim}P)_D]^x}{[(ComE{\sim}P)_D]^x + K_{ComX}{}^x} + vmax_{DprA} * \frac{[ComX_{act}]^d}{[ComX_{act}]^d + K_{DprA}{}^d} + vmax_{SsbB} * \frac{[ComX_{act}]^s}{[ComX_{act}]^s + K_{SsbB}{}^s} + \omega_2 * [ComX_{act}] * [ComZ] - \omega_1 * [ComW] * [ComX_{ina}] - \gamma_{ComX} * [ComX_{ina}]$$
