# Supplementary material for: Dynamic Modeling of Streptococcus pneumoniae Competence Provides Regulatory Mechanistic Insights Into Its Tight Temporal Regulation
Source: Front Microbiol. 2018 Jul 24;9:1637. doi: 10.3389/fmicb.2018.01637 (PMC6066662; doi:10.3389/fmicb.2018.01637)
Supplement: Supplementary file 4 [file Presentation_1.PDF]

Supplementary Methods

Introduction to Petri nets

Petri nets, named after Carl Petri (Petri, 1962) offer a graphical and mathematical formalism adapted to the modeling and analysis of concurrent asynchronous systems (Petri, 1962; Murata, 1989; Chaouiya, 2007; Koch & Heiner, 2008). A basic Petri net is a directed-bipartite graph with two types of nodes: places and transitions. The places represent the resources of the system (proteins in our case) and are passive nodes. Transitions represent the events that can change the state of the resources (reactions in our case) and are active nodes. Places and transitions are connected by directed weighted arcs that describe the causal relation between the resources and the events. Thus, arcs connect only nodes from different types. In a graphical representation, places are depicted by circles, transitions by squares and directed arcs by arrows (Figure 1 below). An arc connect a transition to its pre-conditions, the substrates of a reaction (pre-places), and to its post-conditions, the products of a reaction (post-places). The arc weight sets the number of elements of the resources that are consumed or produced by a transition. They correspond to the stoichiometric coefficients of the reactions. Finally, the tokens are the dynamic elements and can move from one place to another according to the transition that connects both places. The number of tokens in the place indicates the number of available resources (in our case the number of proteins). At any time of the evolution of a Petri net, places contain zero or a positive number of tokens. The distribution of the tokens over the places describes a given state of the system and is called a marking of the Petri net. The initial marking describes the initial state of the system before any event has occurred. The Petri net behavior is defined by firing rules. A transition is enabled and may fire if its preplaces contain at least the number of required tokens that is defined by the weight of the arcs connecting the preplaces to the transition. If the transition fires, the required number of tokens are removed from all its pre-places and the required number of tokens are added to all its post-places, according to the weight of each arc. The movement of the tokens (the “token game”) represents the dynamical evolution of the system.

We used the extended Petri net formalism that includes two other types of arcs: test or read arc and inhibitory arc. A read arc represents a reaction that does not consume its resources (for example, a catalytic reaction where there is no consumption of the enzymes, or in our case the

activation of gene synthesis without consumption of the transcriptional regulator). An inhibitory arc models an inhibition.

Since we studied the dynamics of our network by using ODEs, we did not define any initial marking and did not use the "token game". We used the mathematical formalism of Petri net to check the structural consistency of our model that requires the computation of structural invariants.

Invariant computation

Petri net invariants can be split into place invariants (P-invariants) and transition invariants (T-invariants). A P-invariants corresponds to a set of places over which the sum of tokens is constant and independent of any firing. A T-invariant describes a sequence of transitions (a sub-network) that reproduces an initial state. Starting from an initial state of the system and firing the listed transitions that make up a T-invariant, the system will return to the initial state. Obtaining invariants necessitates the computation of the incidence or reaction matrix C that contains for each transition and each place of the network the difference between the numbers of produced and consumed tokens. It is given by: $C = Post - Pre$, where $Post$ stands for Post-condition matrix and Pre for Pre-condition matrix. The Pre-condition matrix contains the weights of arcs connecting pre-places to transitions and the Post-condition matrix contains the weights of arcs connecting transitions to post-places. A vector of places x is called a P-invariant if it is a non-trivial non-negative integer solution of the linear equation system $x^T.C = 0$ and a vector of transitions y is called a T-invariant if it is a non-trivial non-negative integer solution of the linear equation system $C.y = 0$ (Figure 1 below).

References

Chaouiya C (2007) Petri net modelling of biological networks. *Brief. Bioinform.* **8:** 210–219

Koch I & Heiner M (2008) Petri Nets. In *Wiley Series on Bioinformatics: Computational Techniques and Engineering*, Junker BH & Schreiber F (eds) pp 139–179. Hoboken, NJ, USA: John Wiley & Sons, Inc. Available at: http://doi.wiley.com/10.1002/9780470253489.ch7 [Accessed September 1, 2016]

Murata T (1989) Petri nets: Properties, analysis and applications. *Proc. IEEE* **77:** 541–580

Petri CA (1962) Kommunikation mit Automaten Rhein.-Westfäl. Inst. f. Instrumentelle Mathematik an der Univ. Bonn Available at: https://books.google.fr/books?id=BIGuGwAACAAJ

Petri net graphical representation	Pre-condition matrix (*Pre*)	Post-condition matrix (*Post*)	Incidence matrix *C*
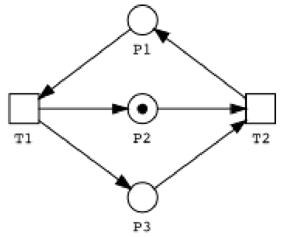	$\begin{bmatrix} -1 & 0 \\ 0 & 1 \\ 0 & 1 \end{bmatrix}$	$\begin{bmatrix} 0 & 1 \\ 0 & 1 \\ 0 & 1 \end{bmatrix}$	$\begin{bmatrix} -1 & 1 \\ 1 & -1 \\ 1 & -1 \end{bmatrix}$

P-invariants	T-invariants
$x^T . C = 0$	$C . y = 0$
system of equations $\begin{cases} -P1 + P2 + P3 = 0 \\ P1 - P2 - P3 = 0 \end{cases}$	system of equations $\begin{cases} -T1 + T2 = 0 \\ T1 - T2 = 0 \\ T1 - T2 = 0 \end{cases}$
non trivial solution $[1,1,0]^T$ and $[1,0,1]^T$	non trivial solution [1,1]

Figure 1. An example of a simple Petri net

Places (P1, P2 and P3) are depicted by circles and transitions T1 and T2 are depicted by squares. Only the place P2 is marked with a token (small black circle). The weight of each arc (arrows) is one, *i.e.*, one token is consumed or produced by each transition. The rows in the pre-condition, post-condition and incidence matrices correspond to the three places in the following order P1, P2 and P3 and the columns to the two transitions T1 and T2. The incidence matrix is used to calculate the P and T-invariants. A vector of places x is a P-invariant if it is a non-trivial non-negative integer solution of the linear equation system $x^T . C = 0$ and a vector of transitions y is a T-invariant if it is a non-trivial non-negative integer solution of the linear equation system $C . y = 0$. In this example, as each place belongs to a P-invariant and each transition to a T-invariant, the model is covered both by P and T-invariants.
