# Supplementary material for: Dynamic Modeling of Streptococcus pneumoniae Competence Provides Regulatory Mechanistic Insights Into Its Tight Temporal Regulation
Source: Front Microbiol. 2018 Jul 24;9:1637. doi: 10.3389/fmicb.2018.01637 (PMC6066662; doi:10.3389/fmicb.2018.01637)
Supplement: Supplementary file 5 [file Image_1.PDF]

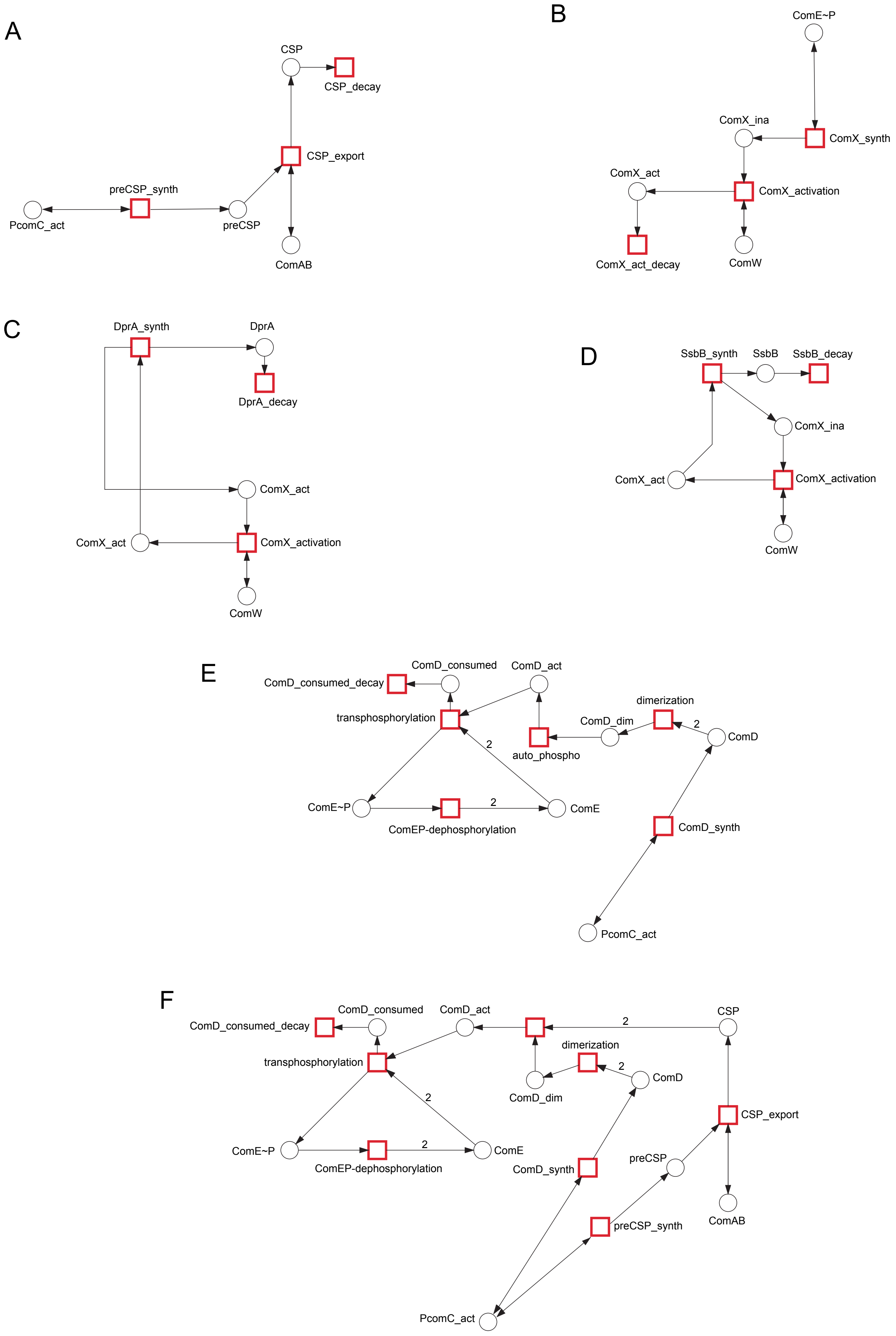

A
CSP
CSP_decay
CSP_export
preCSP_synth
PcomC_act
preCSP
ComAB
B
ComE~P
ComX_ina
ComX_synth
ComX_act
ComX_activation
ComX_act_decay
ComW
C
DprA_synth
DprA
DprA_decay
ComX_act
ComX_act
ComX_activation
ComW
D
SsbB_synth
SsbB
SsbB_decay
ComX_ina
ComX_act
ComX_activation
ComW
E
ComD_consumed
ComD_act
ComD_consumed_decay
transphosphorylation
dimerization
ComD_dim
2
ComD
2
auto_phospho
ComE~P
2
ComE
ComEP-dephosphorylation
ComD_synth
PcomC_act
F
ComD_consumed
ComD_act
CSP
ComD_consumed_decay
2
transphosphorylation
dimerization
2
ComD
ComD_dim
2
CSP_export
2
ComE~P
ComE
ComEP-dephosphorylation
preCSP
ComD_synth
preCSP_synth
ComAB
PcomC_act

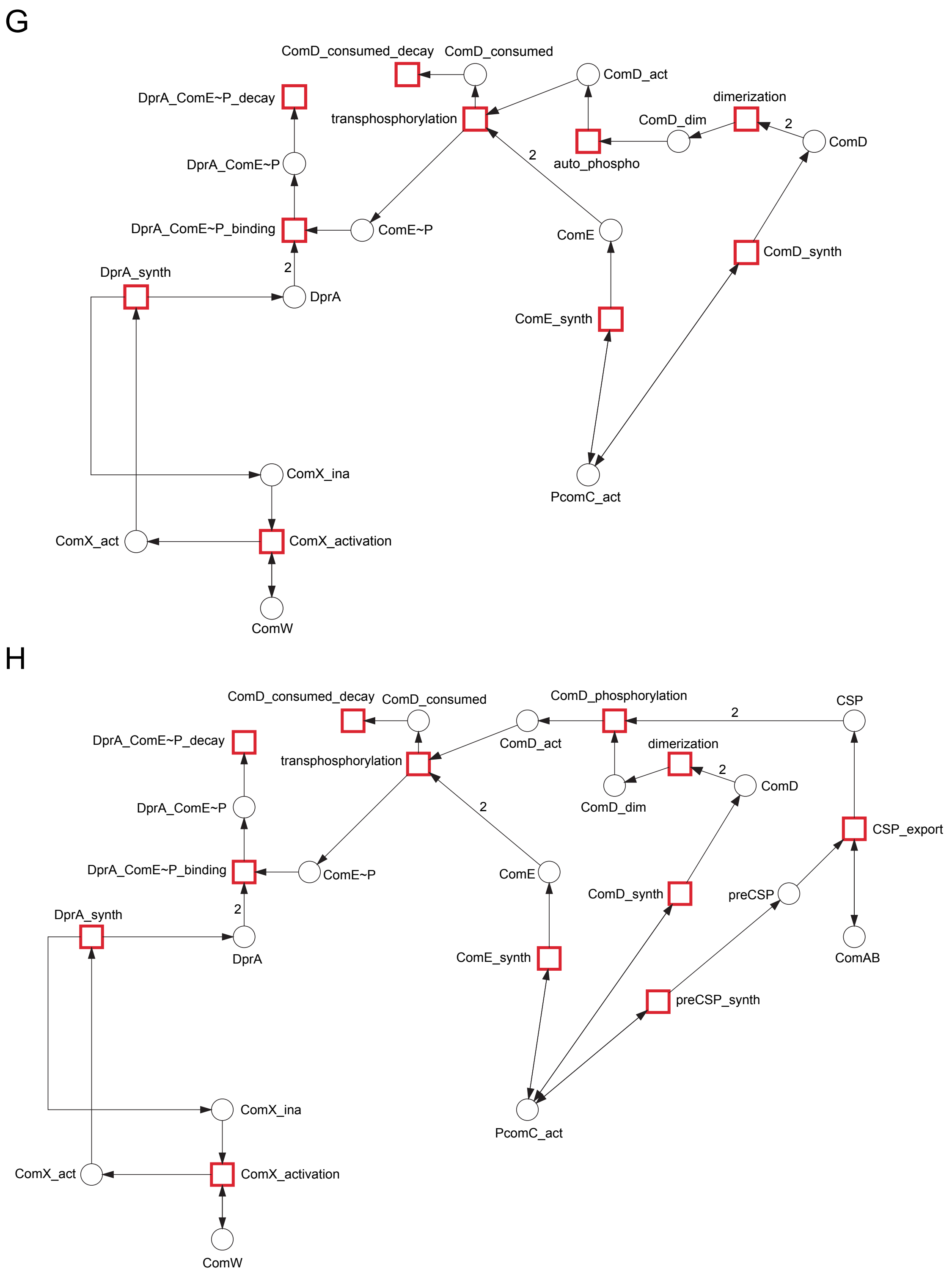

Figure S1 Non-trivial T-invariants of the Petri net model. Only the eight non-trivial T-invariants are shown in panels (A) to (H). Among the trivial T-invariants, six invariants are composed of two transitions representing the synthesis and the degradation of network components (preCSP, ComD, ComE, ComAB, ComW and ComX_ina) and five invariants are composed of two transitions representing the forward and backward directions of reversible reactions (dimerization of ComD and dissociation of the ComD dimer, association and dissociation of ComE and (ComE~P)D from the promoters PcomC and PcomAB (Figure 2).

Transitions (squares) involved in each T-invariant are depicted in red. The weight of the directed arcs connecting places (circles) to transitions, corresponding to the stoichiometric coefficient of the reaction, is indicated only when it is different from one. The biological interpretation of the eight non-trivial T-invariants is: (A) the pre-CSP is synthesized, maturated and exported as CSP that is then degraded; (B) ComX is synthesized in its inactive form and switches to its active form through the interaction of ComXina and ComW; the active form is then degraded; (C) DprA is synthesized and then degraded. As for SsbB, Its synthesis requires the switch from the inactive form of ComX to its active form by ComW interaction; (D) SsbB is synthesized and then degraded. Its synthesis requires the switch from the inactive form of ComX to its active form by ComW interaction; (E) ComD is synthesized, dimerizes and autophosphorylates. The phosphorylated dimer of ComD subsequently phosphorylates its response regulator ComE and is degraded. ComE~P returns to its initial form ComE through dephosphorylation; (F) The only difference with (E) is the phosphorylation of the ComD dimer that requires CSP binding. Thus pre-CSP must be synthesized, matured and exported. The auto_phospho transition will be replaced by the ComD_phosphorylation transition; (G) ComE~P is produced by the transfer to ComE of the phosphoryl group of a dimer of autophosphorylated ComD. The consumed ComD dimer is degraded. ComE~P is sequestrated by DprA synthesized after the switch of ComX from the inactive to the active form by ComW interaction. The complex DprA-ComE~P is then degraded; (H) Series of reactions are the same as described in (G), except that the phosphorylation of the dimer of ComD requires CSP binding and therefore pre-CSP synthesis, maturation and export.
