# Supplementary material for: Dynamic Modeling of Streptococcus pneumoniae Competence Provides Regulatory Mechanistic Insights Into Its Tight Temporal Regulation
Source: Front Microbiol. 2018 Jul 24;9:1637. doi: 10.3389/fmicb.2018.01637 (PMC6066662; doi:10.3389/fmicb.2018.01637)
Supplement: Supplementary file 6 [file Image_2.PDF]

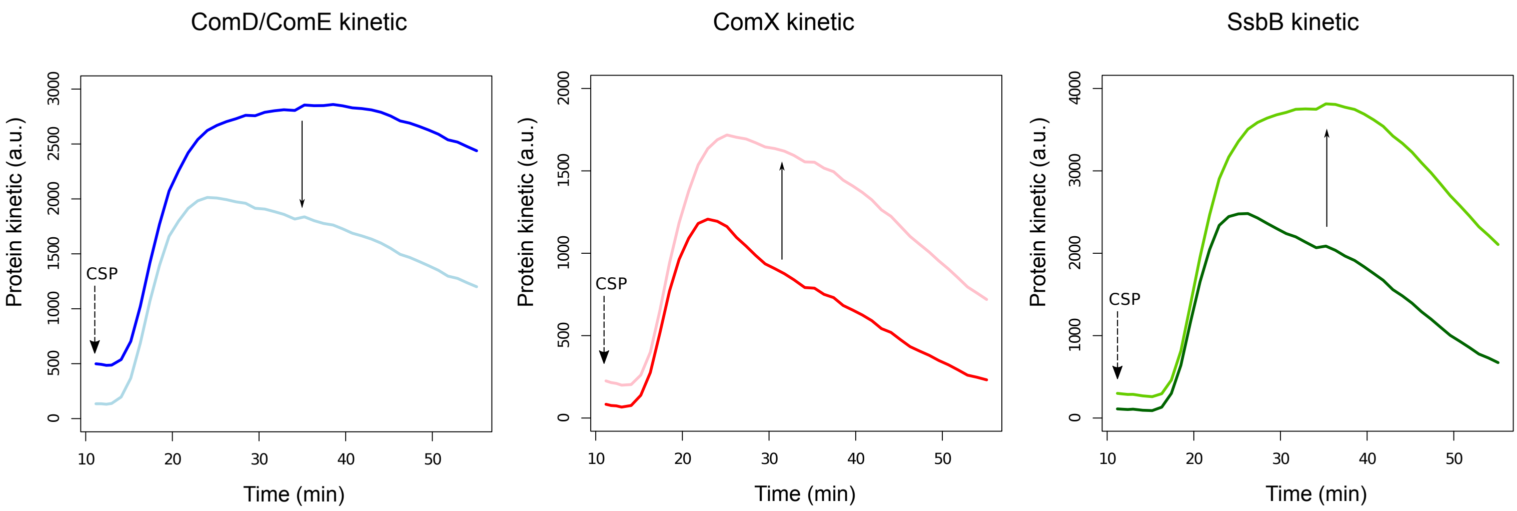

Figure S2 Comparison of the estimation of protein concentration with or without a correction by the luciferase half-life. Dark color lines represent the reconstructed protein concentration kinetics by taking into account only protein life time. Light color lines correspond to reconstructed protein kinetics corrected for differences in half-lives between luciferase and the protein whose gene activity is measured using the equation proposed by Stefan and collaborators (Stefan *et al.*, 2015). The protein life times used for computation are: 8 min for ComX and SsbB, 80 min for ComD/ComE and 21.6 min for luciferase.
