# Supplementary material for: Dynamic Modeling of Streptococcus pneumoniae Competence Provides Regulatory Mechanistic Insights Into Its Tight Temporal Regulation
Source: Front Microbiol. 2018 Jul 24;9:1637. doi: 10.3389/fmicb.2018.01637 (PMC6066662; doi:10.3389/fmicb.2018.01637)
Supplement: Supplementary file 7 [file Image_3.PDF]

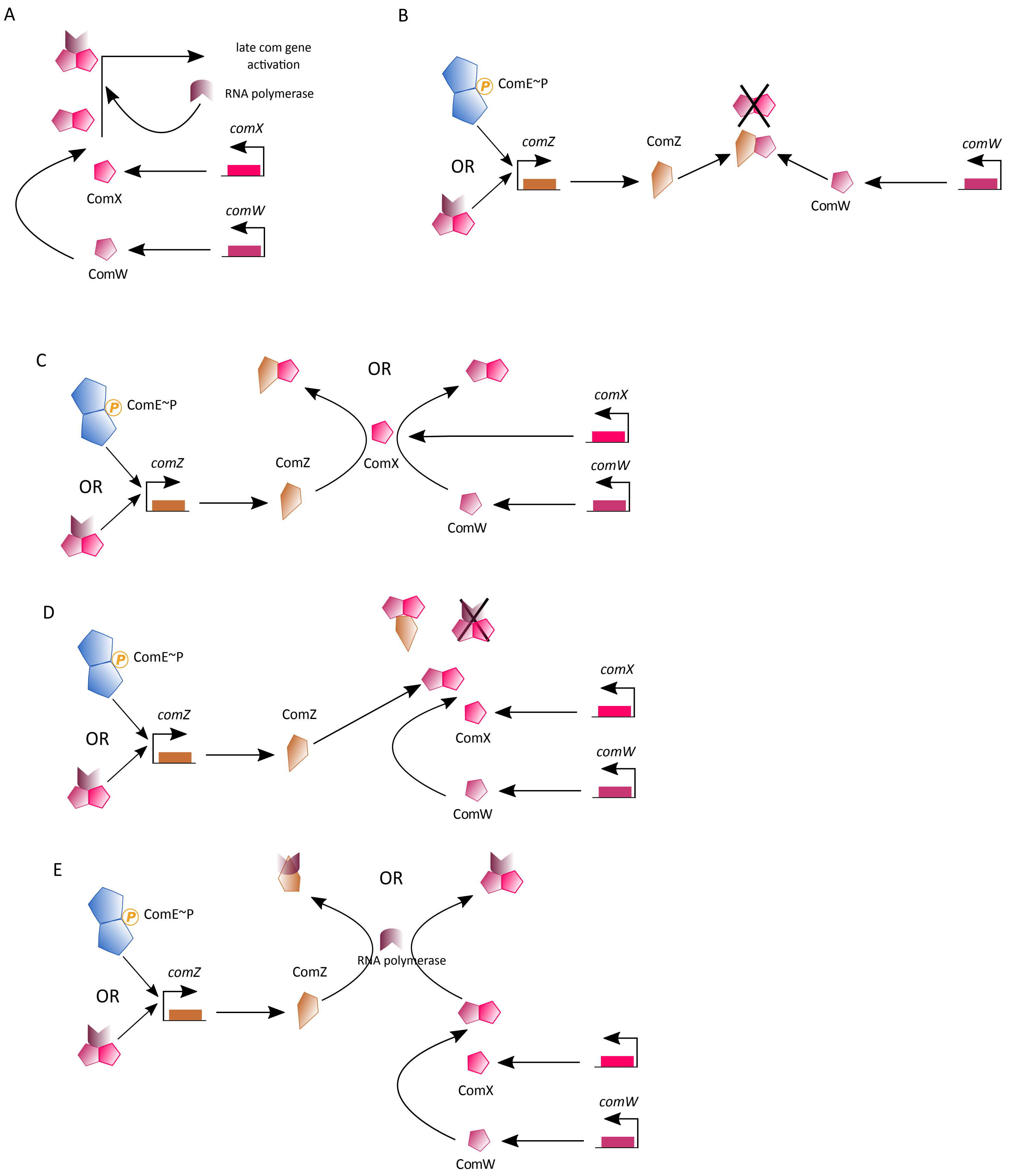

Figure S3. Schematic diagrams of alternative hypothetical models for competence shut-off involving an additional unknown actor ComZ. (A) Part extracted from figure 1 depicting the current knowledge on ComX activation that was modeled in our initial network where ComW interacts with ComX to improve its binding to core RNA polymerase. (B) ComZ interacts with ComW preventing the binding between ComW and ComX and the formation of the active form of ComX. (C) ComZ competes with ComW for binding to the inactive form of ComX and affects the formation of the active form of ComX. (D) ComZ binds to the active form of ComX and inhibitis its σ factor function. (E) ComZ and the active form of ComX compete for binding to core RNA polymerase. In this latter model, ComZ will correspond to the housekeeping σA factor. In the four alternative hypotheses for ComZ action, its gene can be either an early *com* gene which expression is under the control of ComE~P or a late *com* gene which sysnthesis depends on ComX. The same symbolisms as in figure 1 are used.
