# Supplementary material for: Dynamic Modeling of Streptococcus pneumoniae Competence Provides Regulatory Mechanistic Insights Into Its Tight Temporal Regulation
Source: Front Microbiol. 2018 Jul 24;9:1637. doi: 10.3389/fmicb.2018.01637 (PMC6066662; doi:10.3389/fmicb.2018.01637)
Supplement: Supplementary file 8 [file Image_4.PDF]

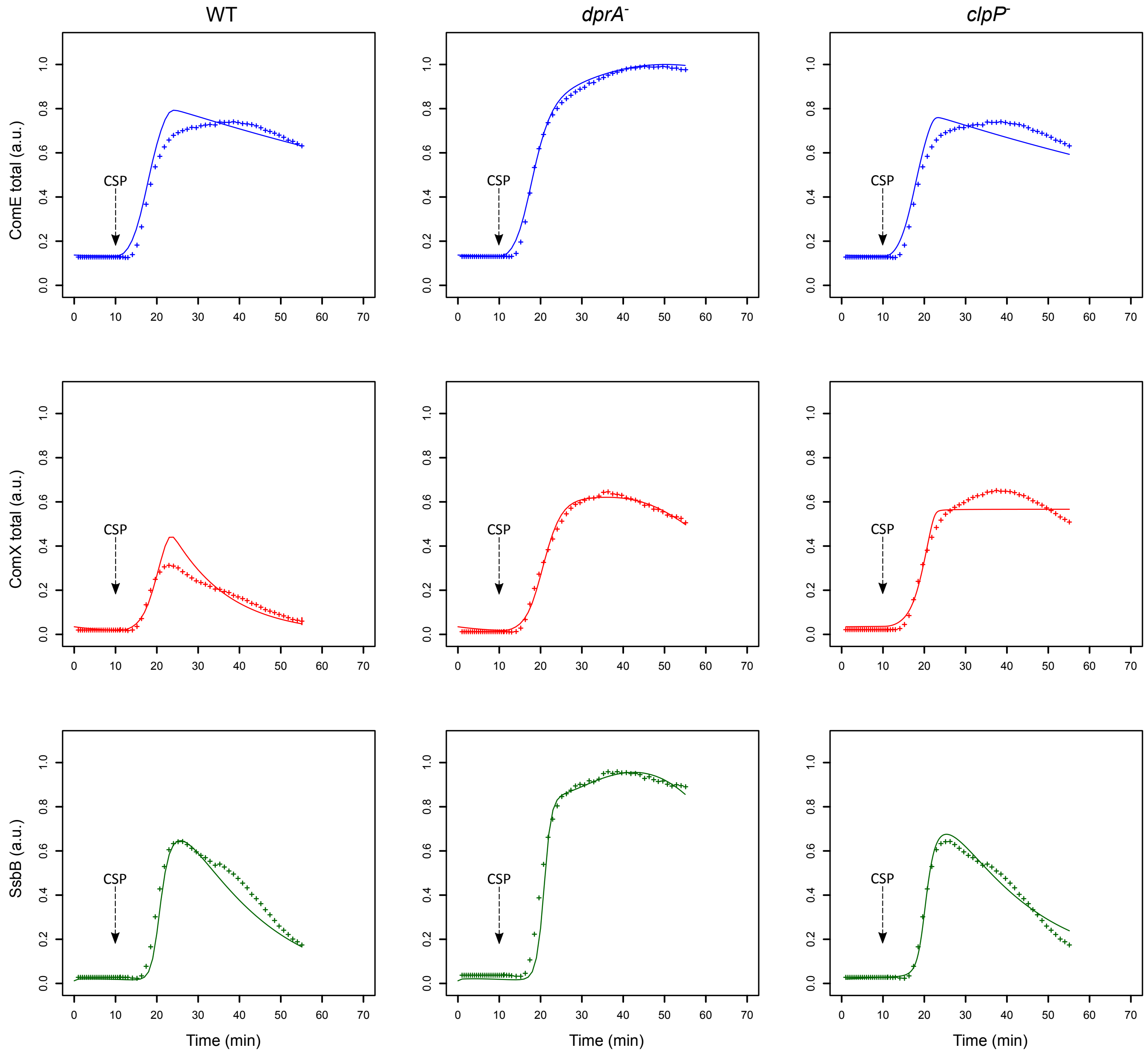

Figure S4. Comparison of the experimental and simulated protein kinetics obtained with the model where a late gene product ComZ and the active form of ComX compete for binding to RNA polymerase. Comparison of simulated data with the experimental measurements are shown for the WT strain, the *dprA* mutant strain and the *clpP* mutant strain. Experimental and simulated data are represented by crosses and solid lines respectively. In the *dprA* mutant the simulated protein kinetics were obtained by setting the DprA maximal synthesis rate ($vmax_{dprA}$) to 0. In the *clpP* mutant both ComX (γ_{ComX}) and ComW (γ_{ComW}) degradation constants were designated as 0. Competence gene expression was assumed to be at steady-state at the beginning of the simulation, and competence development was induced by adding one arbitrary unit (a.u.) of CSP (corresponding to 100 ng/mL) at t = 10 min in order to reproduce the experimental protocol (100 ng/mL added after 10 min incubation (Mirouze *et al.*, 2013)). The *comD* and *comE* genes being in an operon, the blue line represents both $ComD_{total}$ and $ComE_{total}$ kinetics. Red and green lines depict $ComX_{total}$ and SsbB kinetics respectively. Protein concentrations have been normalized with respect to the maximum values obtained over all computed data sets; therefore values are given in arbitrary units (a.u.).
