# Supplementary material for: Dynamic Modeling of Streptococcus pneumoniae Competence Provides Regulatory Mechanistic Insights Into Its Tight Temporal Regulation
Source: Front Microbiol. 2018 Jul 24;9:1637. doi: 10.3389/fmicb.2018.01637 (PMC6066662; doi:10.3389/fmicb.2018.01637)
Supplement: Supplementary file 12 [file Image_8.PDF]

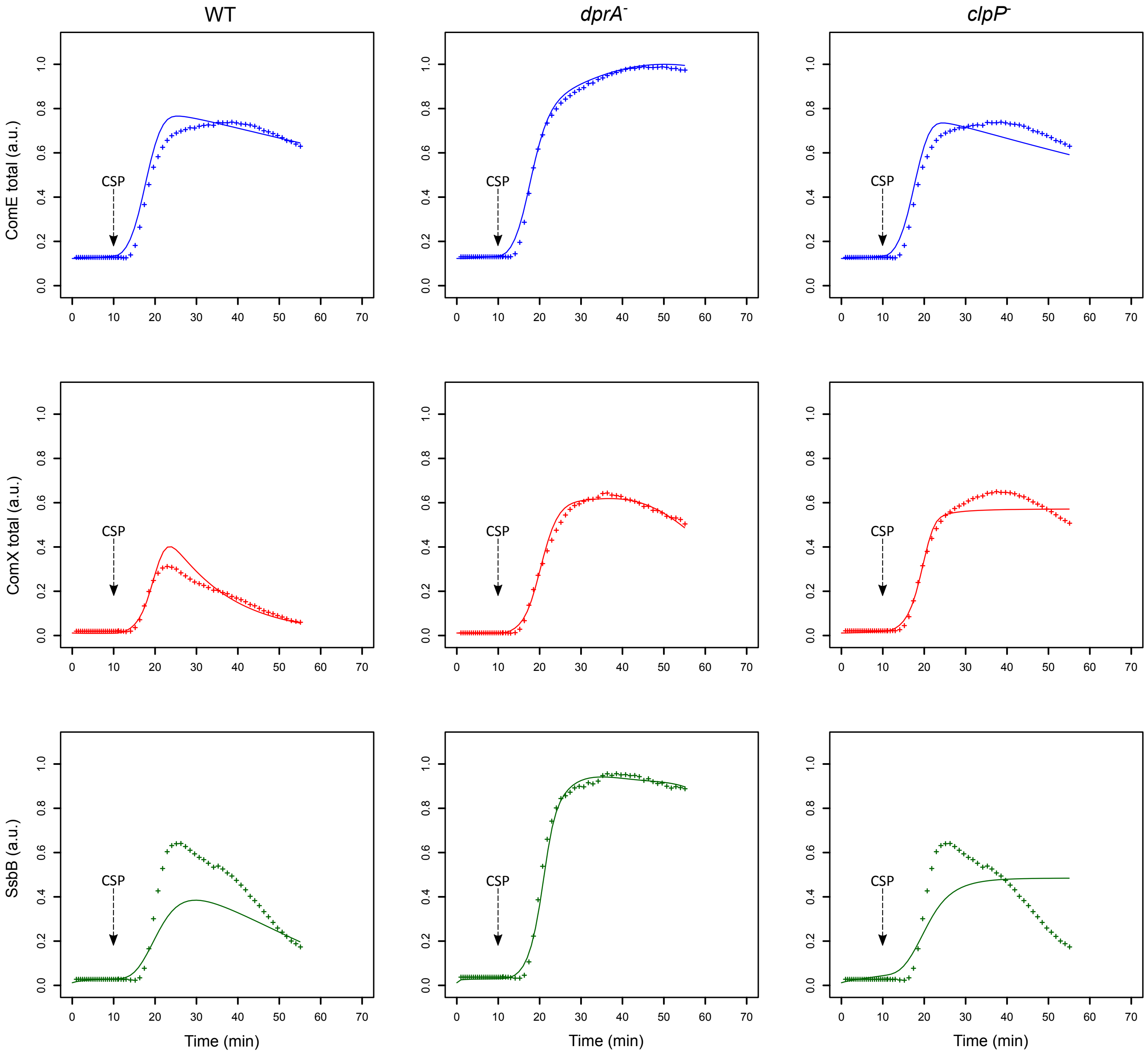

Figure S8. Comparison of the experimental and simulated protein kinetics obtained with the model where an early gene product ComZ and ComW compete for interacting with the inactive form of ComX. Comparison of simulated data with the experimental measurements are shown for the WT strain, the *dprA* mutant strain and the *clpP* mutant strain. Symbolisms and color code are the same as in Figure S4 as well as the simulated protocol.
