# Supplementary material for: Dynamic Modeling of Streptococcus pneumoniae Competence Provides Regulatory Mechanistic Insights Into Its Tight Temporal Regulation
Source: Front Microbiol. 2018 Jul 24;9:1637. doi: 10.3389/fmicb.2018.01637 (PMC6066662; doi:10.3389/fmicb.2018.01637)
Supplement: Supplementary file 14 [file Image_10.PDF]

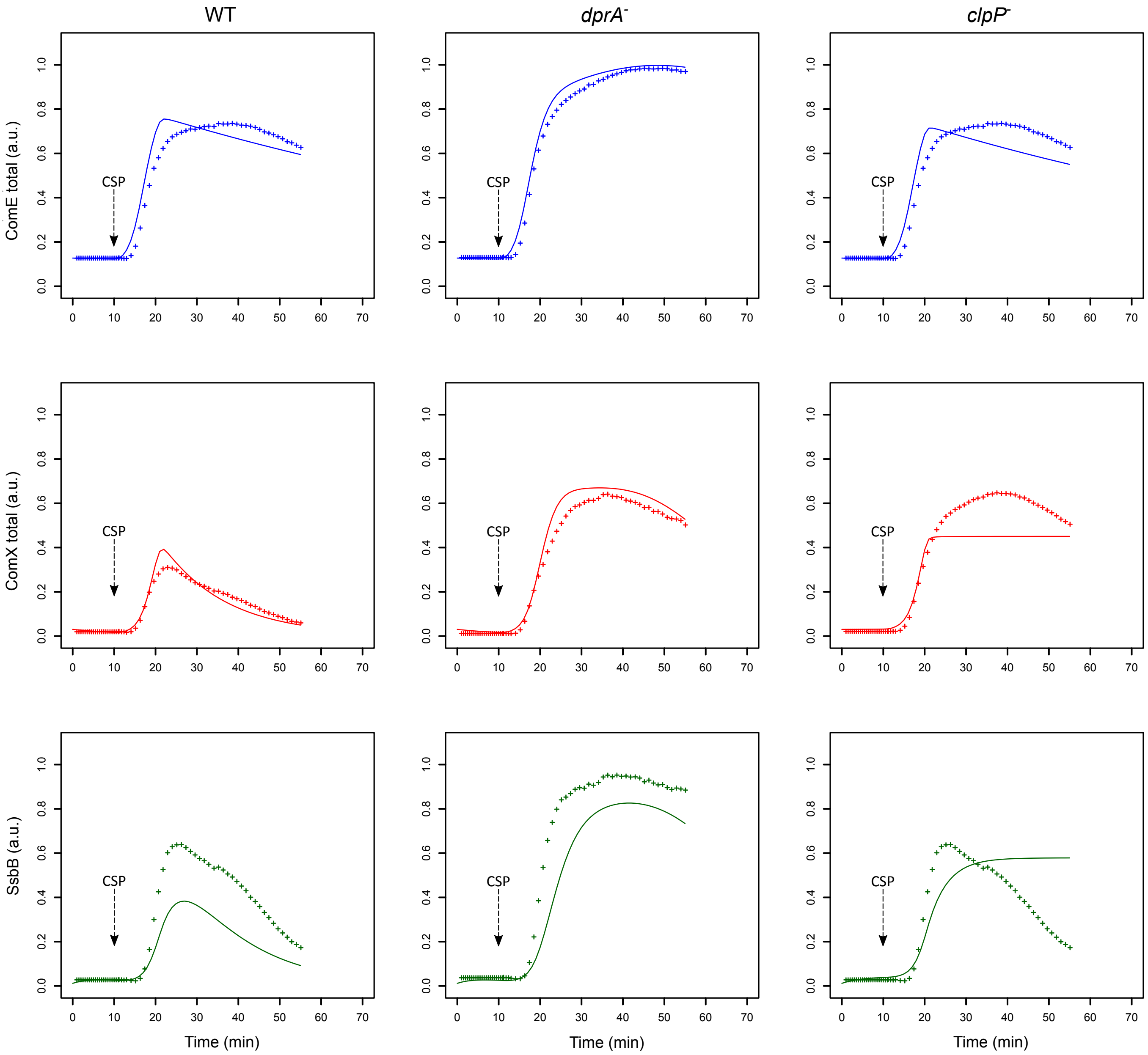


**Figure S10. Comparison of the experimental and simulated protein kinetics obtained with the model where an early gene product ComZ inactivates the active form of ComX.** Comparison of simulated data with the experimental measurements are shown for the WT strain, the *dprA* mutant strain and the *clpP* mutant strain. Symbolisms and color code are the same as in Figure S4 as well as the simulated protocol.
