# Supplementary material for: Dynamic Modeling of Streptococcus pneumoniae Competence Provides Regulatory Mechanistic Insights Into Its Tight Temporal Regulation
Source: Front Microbiol. 2018 Jul 24;9:1637. doi: 10.3389/fmicb.2018.01637 (PMC6066662; doi:10.3389/fmicb.2018.01637)
Supplement: Supplementary file 15 [file Image_11.PDF]

A

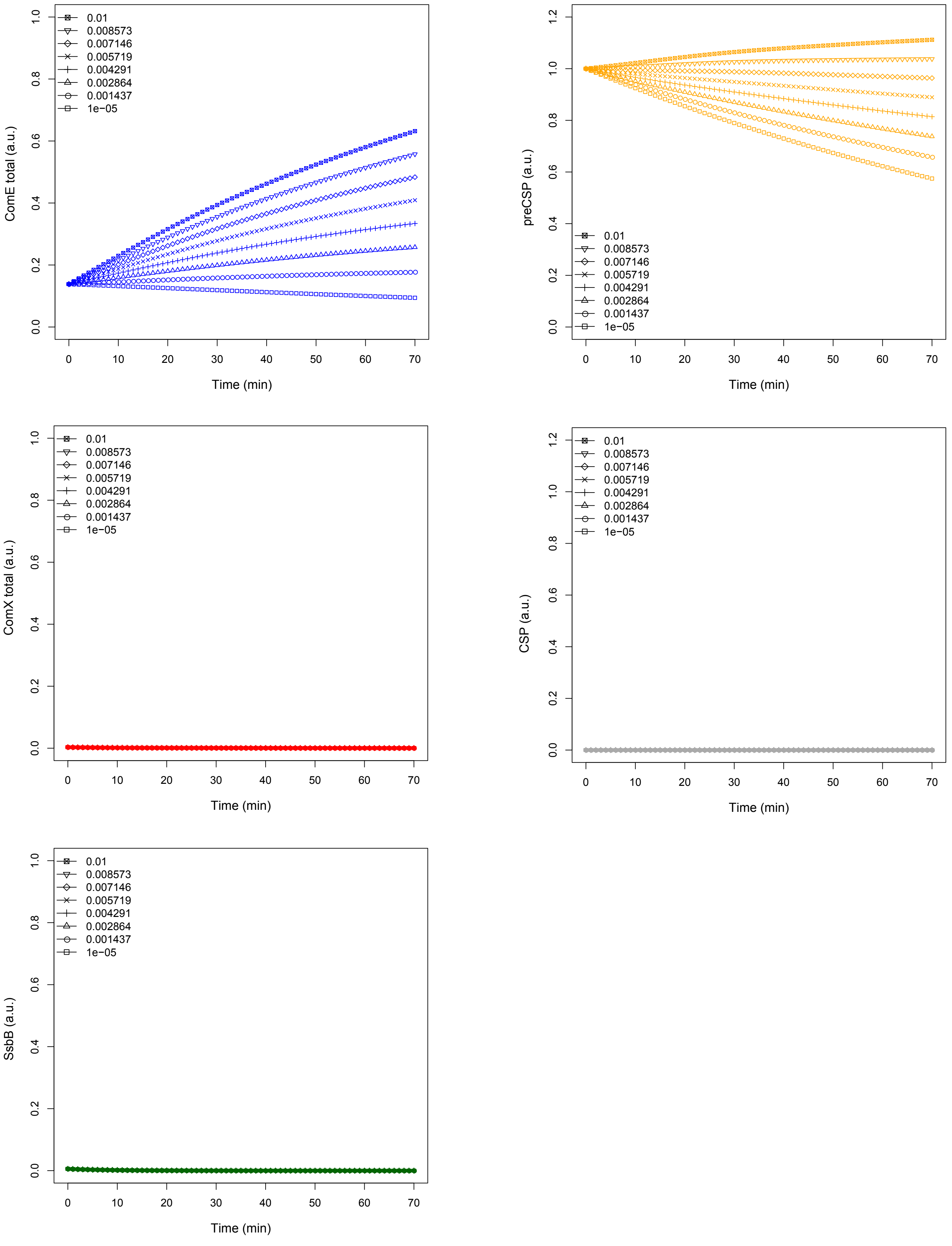
ComE total (a.u.)
preCSP (a.u.)
ComX total (a.u.)
CSP (a.u.)
SsbB (a.u.)
Time (min)
0.01
0.008573
0.007146
0.005719
0.004291
0.002864
0.001437
1e−05

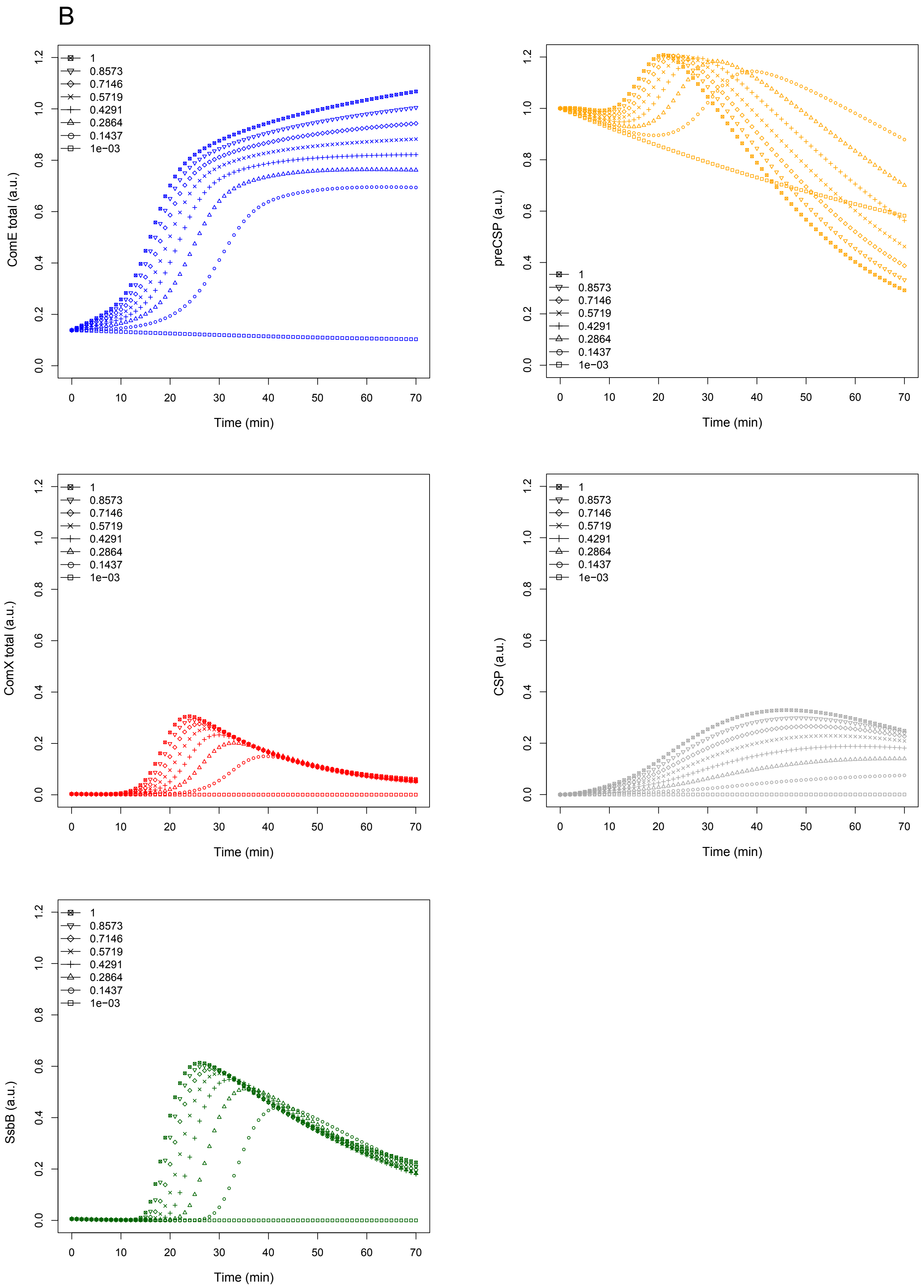

Figure S11. Both basal synthesis rates of *comAB* and *comCDE* influence spontaneous competence development.
(A) The simulated network dynamics is followed without external CSP addition for a ComCDE basal synthesis rate (β_{comCDE}) varying from 10^{-5} to 10^{-2} a.u. min^{-1} without any modification of the other parameter values of the model. $ComE_{total}$, $ComX_{total}$ and SsbB kinetics are shown using the same color code as in Figure 3. Pre-CSP (yellow) and CSP (grey) kinetics are also reported. (B) The same simulations as in (A) are performed but β_{comAB} varies conjointly with β_{comCDE} with $\beta_{comAB} = 100 * \beta_{comCDE}$.
