# Supplementary material for: Dynamic Modeling of Streptococcus pneumoniae Competence Provides Regulatory Mechanistic Insights Into Its Tight Temporal Regulation
Source: Front Microbiol. 2018 Jul 24;9:1637. doi: 10.3389/fmicb.2018.01637 (PMC6066662; doi:10.3389/fmicb.2018.01637)
Supplement: Supplementary file 16 [file Image_12.PDF]

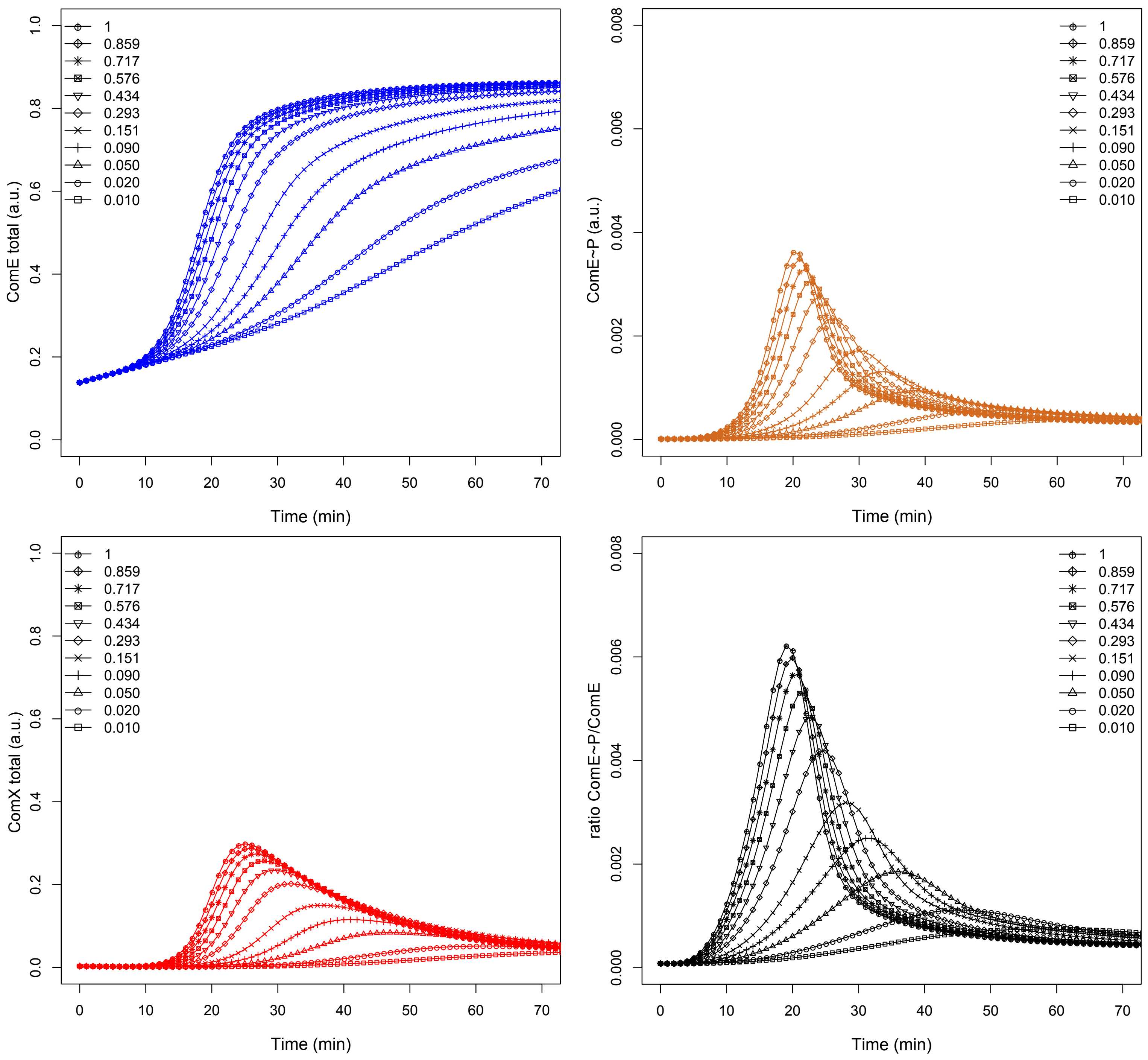

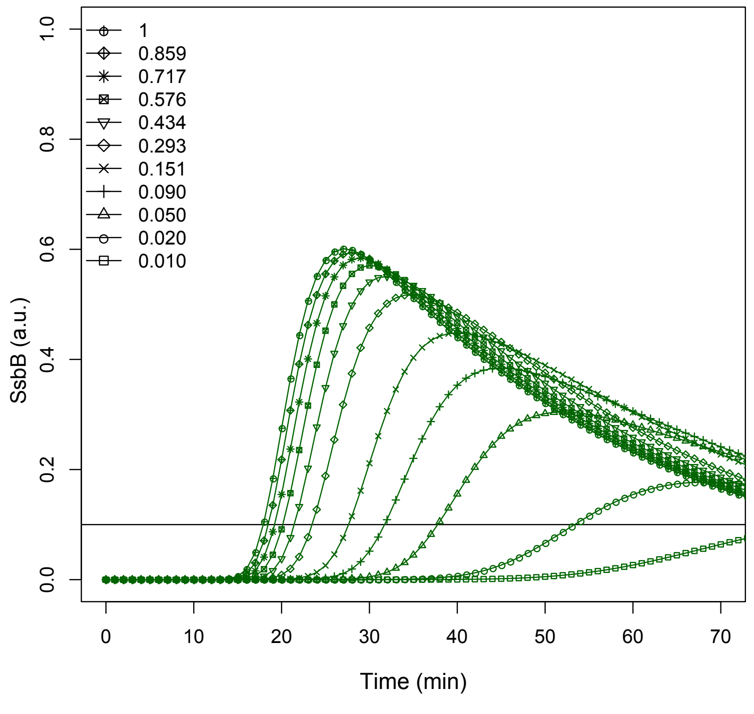

Figure S12. The ratio ComE~P/ComE is important for spontaneous competence development. The simulated network dynamics is followed without external CSP addition for a fixed value of the basal synthesis rate of ComCDE (0.005 a.u. min^{-1}) and a basal synthesis rate of ComAB varying from 0.01 to 1 a.u. min^{-1}. $ComE_{total}$, $ComX_{total}$ and SsbB kinetics are shown using the same color code as in Figure 3. The ComE~P kinetics are shown in brown and the ratio ComE~P/ ComE is depicted in black. The horizontal black line on the SsbB panel corresponds to the threshold of 0.1 a.u.
