# Supplementary material for: Dynamic Modeling of Streptococcus pneumoniae Competence Provides Regulatory Mechanistic Insights Into Its Tight Temporal Regulation
Source: Front Microbiol. 2018 Jul 24;9:1637. doi: 10.3389/fmicb.2018.01637 (PMC6066662; doi:10.3389/fmicb.2018.01637)
Supplement: Supplementary file 17 [file Image_13.PDF]

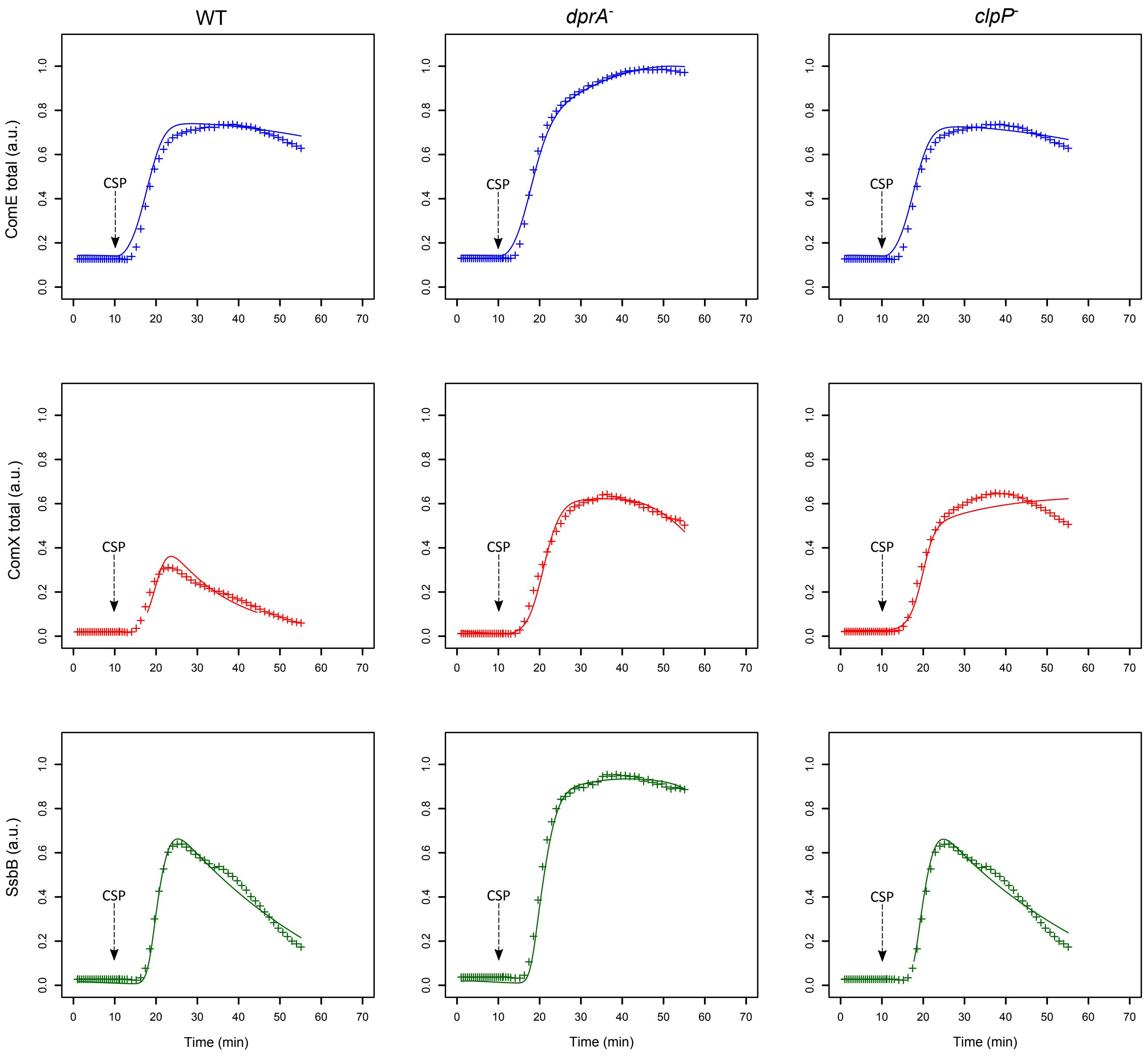

Figure S13. Comparison of the experimental and simulated protein kinetics obtained with the model where a late gene product ComZ interacts with ComW and competes with the active form of ComX for binding to RNA polymerase. Comparison of simulated data with the experimental measurements are shown for the WT strain, the *dprA* mutant strain and the *clpP* mutant strain. Symbolisms and color code are the same as in Figure S4 as well as the simulated protocol.
